# Venetoclax triggers sublethal apoptotic signaling in venetoclax-resistant acute myeloid leukemia cells and induces vulnerability to PARP inhibition and azacitidine

**DOI:** 10.1038/s41419-024-07140-4

**Published:** 2024-10-16

**Authors:** Mahesh Tambe, Sarah Unterberger, Mette C. Kriegbaum, Ida Vänttinen, Ezgi June Olgac, Markus Vähä-Koskela, Mika Kontro, Krister Wennerberg, Caroline A. Heckman

**Affiliations:** 1 Institute for Molecular Medicine Finland (FIMM), Helsinki Institute of Life Science (HiLIFE), iCAN Digital Precision Cancer Medicine Flagship, University of Helsinki, Helsinki, Finland; 2 Biotech Research & Innovation Centre (BRIC), University of Copenhagen, Copenhagen, Denmark; 3 Department of Hematology, Helsinki University Central Hospital Comprehensive Cancer Center, Helsinki, Finland; 4 Foundation for the Finnish Cancer Institute, Helsinki, Finland

**Keywords:** Acute myeloid leukaemia, Acute myeloid leukaemia

## Abstract

Venetoclax plus azacitidine treatment is clinically beneficial for elderly and unfit acute myeloid leukemia (AML) patients. However, the treatment is rarely curative, and relapse due to resistant disease eventually emerges. Since no current clinically feasible treatments are known to be effective at the state of acquired venetoclax resistance, this is becoming a major challenge in AML treatment. Studying venetoclax-resistant AML cell lines, we observed that venetoclax induced sublethal apoptotic signaling and DNA damage even though cell survival and growth were unaffected. This effect could be due to venetoclax inducing a sublethal degree of mitochondrial outer membrane permeabilization. Based on these results, we hypothesized that the sublethal apoptotic signaling induced by venetoclax could constitute a vulnerability in venetoclax-resistant AML cells. This was supported by screens with a broad collection of drugs, where we observed a synergistic effect between venetoclax and PARP inhibition in venetoclax-resistant cells. Additionally, the venetoclax-PARP inhibitor combination prevented the acquisition of venetoclax resistance in treatment naïve AML cell lines. Furthermore, the addition of azacitidine to the venetoclax-PARP inhibitor combination enhanced venetoclax induced DNA damage and exhibited exceptional sensitivity and long-term responses in the venetoclax-resistant AML cell lines and samples from AML patients that had clinically relapsed under venetoclax-azacitidine therapy. In conclusion, we mechanistically identify a new vulnerability in acquired venetoclax-resistant AML cells and identify PARP inhibition as a potential therapeutic approach to overcome acquired venetoclax resistance in AML.

## Introduction

Treatment with the BCL2 inhibitor venetoclax in combination with a hypomethylating agent (azacitidine or decitabine) in elderly and unfit acute myeloid leukemia (AML) patients leads to high remission rates and significantly extends overall survival over the previously available treatment regimens [1, 2]. However, despite the clinical benefits, the disease typically recurs over time, and at that point, no effective treatments are known [3]. Novel treatment options are therefore required to provide long-term benefits for venetoclax-azacitidine relapsing patients.

The anti-cancer effect of venetoclax is associated with its ability to antagonize the anti-apoptotic function of BCL2, resulting in mitochondrial outer membrane permeabilization (MOMP) and release of cytochrome C into the cytoplasm. Cytoplasmic cytochrome C triggers caspase activation and induces apoptosis signaling that consequently leads to apoptotic cell death [4]. However, induction of MOMP is not necessarily a commitment to cell death, as cells undergoing sublethal MOMP may survive [5, 6]. Sublethal MOMP occurs when only a fraction of mitochondria in a cell undergoes MOMP (minority MOMP) or a few unprimed mitochondria in a cell repopulate (incomplete MOMP), which may result in cell survival [5–7]. Sublethal MOMP may lead to limited caspase activity and DNA damage that is inadequate to induce apoptotic cell death [5–7].

Poly (ADP-ribose) polymerase (PARP) enzymes are critical for initial response to DNA damage. PARP is recruited to the sites of DNA damage where it promotes the recruitment of additional DNA repair proteins [8, 9]. PARP inhibitors (PARPi) have been successfully used in clinics to treat subsets of ovarian, breast, prostate, and pancreatic cancers. PARPi have failed as single-agent therapies in AML [8, 9], but in combination with a hypomethylating agent (azacitidine or decitabine), PARPi has shown promising anti-leukemic effects in preclinical studies [10]. However, in a clinical trial setting, the patients with relapsed or refractory AML considered unfit for intensive chemotherapy showed poor response to the combination of decitabine and PARPi [11].

In this study, we show that venetoclax triggers sublethal MOMP, sublethal apoptotic signaling, and DNA damage in venetoclax-resistant AML cell lines. This observation led us to test the combination of venetoclax, hypomethylating agents, and PARPi on venetoclax-resistant AML cell lines and primary AML patient samples that had clinically relapsed under venetoclax-azacitidine therapy. The long-term cell growth assays revealed exceptional sensitivity of the triple combination of venetoclax, hypomethylating agents, and PARPi in our model systems.

## Results

**Fig. 1:**
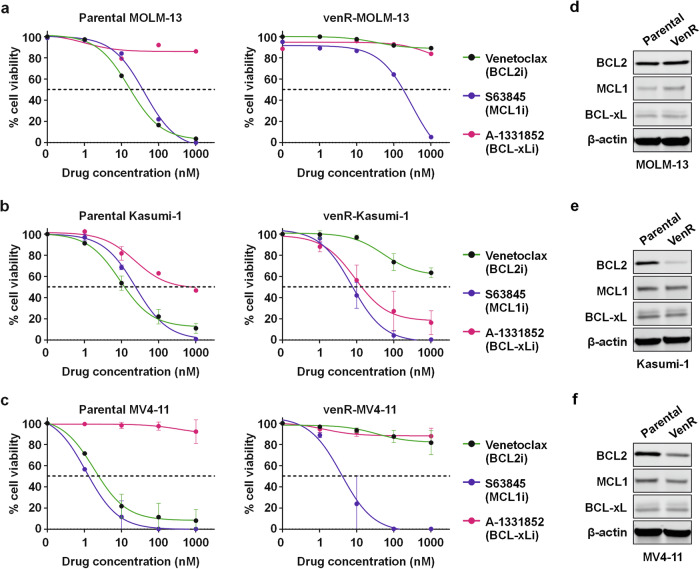
Characterization of venetoclax-resistant AML cell lines. Percentage cell viability of **a** MOLM-13 cells (parental and venR), **b** Kasumi-1 cells (parental and venR), and **c** MV4-11 cells (parental and venR) in response to venetoclax (BCL2i), S63845 (MCL1i) and A-1331852 (BCL-xLi) treated at the indicated concentrations. Cell viability was measured after 72 h of drug treatment using the CellTiter-Glo assay. The data is mean ± s.d. from 2 to 3 independent experiments. **d**–**f** The immunoblots showing BCL2, MCL1, and BCL-xL proteins in **d** MOLM-13 (parental and venR cells), **e** Kasumi-1 (parental and venR cells), and **f** MV4-11 (parental and venR cells) normalized to β-actin.

We aimed to gain a deeper understanding of the cell states of venR AML with the goal of identifying novel vulnerabilities in these cells. We generated venR-AML cell lines (venR-MOLM-13, venR-Kasumi-1, and venR-MV4-11), which exhibited drastically reduced sensitivity to venetoclax (>100-fold) but retained their sensitivity to MCL1 inhibition, as compared to their parental counterparts (Fig. 1a–c). Moreover, the venR-Kasumi-1 cells exhibited increased sensitivity to BCL-xL inhibition (Fig. 1a–c). Compared to their parental counterparts, venR-MOLM-13 cells showed increased levels of MCL1, while both venR-Kasumi-1 and venR-MV4-11 showed decreased BCL2 levels (Fig. 1d–f, Supplementary Fig. 1).

**Fig. 2:**
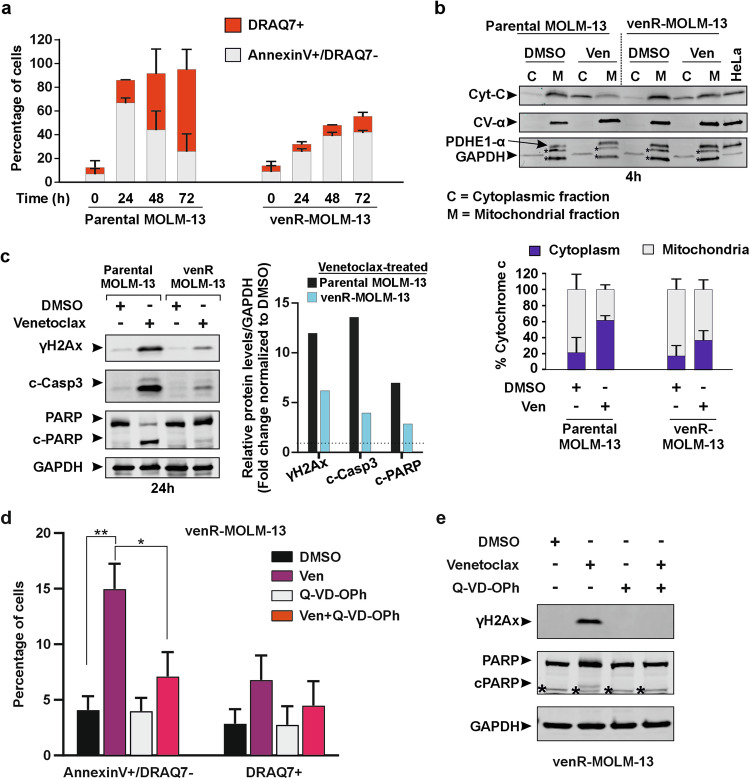
Venetoclax induces sublethal MOMP and caspase-dependent sublethal apoptotic signaling and DNA damage in venetoclax-resistant AML cells. **a** Percentage of Annexin V+/DRAQ7− and DRAQ7+ (Annexin V+/DRAQ7+ and Annexin V−/DRAQ7+) cells. The parental MOLM-13 and venR-MOLM-13 cells were treated with venetoclax (100 nM) for the indicated times. The data are visualized as mean ± s.d. from independent experiments (parental cells = 2 experiments, venR cells = 3 experiments). **b** Immunoblots and the corresponding quantification of percentage of cytochrome C (Cyt-C) in cytoplasmic and mitochondrial cell fractions of parental MOLM-13 and venR-MOLM-13 cells treated with DMSO or 100 nM venetoclax (Ven) for 4 h. HeLa cell lysate served as a positive control for the detection of mitochondrial markers CV-α and PDHE1-α, and cytoplasmic marker GAPDH. The bands indicated with * are non-specific bands. The data is mean ± s.d. from two independent experiments, and one corresponding immunoblot is shown. **c** The parental MOLM-13 and venR-MOLM-13 cells were treated with DMSO or 100 nM venetoclax for 24 h. Immunoblots showing the expression of γH2Ax, cleaved-caspase3 (c-Casp3), PARP (non-cleaved), c-PARP (cleaved-PARP), and GAPDH in parental MOLM-13 and venR-MOLM-13 cells. The graph shows a fold change of relative protein levels normalized to GAPDH and DMSO control (dotted line). **d**, **e** The venR-cells were treated with venetoclax and Q-VD-OPh as single agents and in combination for 24 h. **d** The graph shows percentage of Annexin V+/DRAQ7− and DRAQ7+ (Annexin V+/DRAQ7+ and Annexin V-/DRAQ7+) cells. Data were compared using a student’s *t*-test (two-tailed unpaired) to determine statistical significance such that *p* ≤ 0.05 = * and *p* ≤ 0.01 = **. **e** The immunoblot shows expression of γH2Ax, PARP, cPARP (cleaved-PARP), and GAPDH. The non-specific bands are indicated with *. The data is mean ± s.d. from three independent experiments.

We treated parental MOLM-13 and venR-MOLM-13 cells with 100 nM venetoclax and stained the cells for Annexin V (marker for apoptosis) and DRAQ7 (marker for dead cells) (Supplementary Fig. 2a). As expected, parental MOLM-13 cells exhibited an early increase in Annexin V positivity (Annexin V+/DRAQ7− cells) followed by an induction of cell death (DRAQ7+ cells) (Fig. 2a, Supplementary Fig. 2b). The venR-MOLM-13 cells, on the other hand, showed a delayed but clear induction of Annexin V positivity in a subset of cells, but this did not lead to large-scale cell death (Fig. 2a, Supplementary Fig. 2c). To investigate Annexin V induction at the single cell level in the venR-MOLM-13 cells and how this impacted the ability of cells to proliferate, we treated venR-MOLM-13 cells with venetoclax for 48 h after which the cells were stained with Annexin V. To understand whether low- versus high-annexin V induced cells were able to promote proliferation of the venetoclax-treated cell population, four cell fractions (P1–P4) were sorted such that P1 < P2 < P3 < P4 for Annexin V signal intensity (Supplementary Fig. 3a). The fate of untreated cells and sorted cell populations P1–P4 were evaluated in cell proliferation and colony formation assays. We observed that the venetoclax-treated Annexin V-negative P1 and weakly Annexin V-positive P2 cell populations were highly viable and proliferated, similar to untreated venR-MOLM-13 cells (Supplementary Fig. 3b–d). The proliferative capacity of the moderately Annexin V-stained P3 population dropped to 60% on day 2 after sorting, while yielding half the number of colonies on day 7 compared to untreated cells and to the Annexin V-negative P1 and weakly Annexin V-positive P2 populations (Supplementary Fig. 3b–d). Correspondingly, cell viability and colony formation capacity were lowest in the highest Annexin V-positive P4 population, although even this fraction contained live surviving cells capable of resuming growth. These results indicate that most of the proliferation of the venetoclax-treated venR-MOLM-13 culture happens in cells that have only responded with a weak to moderate Annexin V induction, but some cells with a high Annexin V response can also continue to proliferate, suggesting different levels of sublethal apoptotic signaling [7]. The sublethal apoptotic signaling in venetoclax-treated venR-MOLM-13 cells was also confirmed by live cell imaging. The parental and venR-MOLM-13 cells were treated with DMSO, venetoclax, or S63845 (MCL1 inhibitor). In addition, the cell growth medium was supplemented with caspase-3/7 green dye (a marker for apoptosis) and cytotox red dye (a marker for cell death). In parental MOLM-13 cells, venetoclax and S63845 treatment induced rapid caspase-3/7 activation followed by large-scale cell death (Supplementary Fig. 4a–c). On the other hand, in venR-MOLM-13 cells, venetoclax treatment induced delayed caspase-3/7 activation without a considerable increase in cell death, while S63845 induced rapid caspase-3/7 activation accompanied by large-scale cell death (Supplementary Fig. 4a–c). These results indicate that venetoclax induces sublethal apoptotic signaling in venR-MOLM-13 cells that does not translate into cell death.

Next, we investigated the extent of MOMP induction in parental MOLM-13 and venR-MOLM-13 cells in response to venetoclax. The translocation of cytochrome C was used as a marker for MOMP induction. First, by Western blotting, we detected a similar level of cytochrome C in the cytoplasm (~20%) and mitochondria (~80%) of control-treated parental MOLM-13 and venR-MOLM-13 cells (Fig. 2b). Venetoclax treatment induced cytochrome C translocation from mitochondria to the cytoplasm such that after 4 h venetoclax treatment 61 and 36% of cytochrome C was cytoplasmic in the parental MOLM-13 and venR-MOLM-13 cells, respectively (Fig. 2b). Further, we measured loss of mitochondrial cytochrome C at the single cell level in live cells by flow cytometry after 4 and 24 h of venetoclax treatment. This allowed us to categorize cells into those undergoing widespread MOMP (cytochrome C negative cells) or sublethal MOMP (cells with reduced mitochondrial cytochrome C) (Supplementary Fig. 5a). Importantly, dead cells that stained positive for the viability dye ef780 were excluded from the analysis. Venetoclax treatment induced significant widespread MOMP in parental MOLM-13 cells after both 4 and 24 h venetoclax treatment, whereas in venR-MOLM-13 cells, there was no significant increase in cells with widespread MOMP after venetoclax treatment (Supplementary Fig. 5b, c). Importantly, a peak shift of the cytochrome C-positive cell population was observed (indicated by arrows) in both the parental and venR-MOLM-13 cells indicating a population-wide sublethal MOMP response (Supplementary Fig. 5b). A significant sublethal MOMP shift was already induced after 4 h and was also present at 24 h post venetoclax treatment in parental MOLM-13 cells, whereas in venR-MOLM-13 cells, a significant sublethal MOMP shift was observed only after 24 h venetoclax treatment (Supplementary Fig. 5b, d). Together, the findings in Fig. 2a, b and Supplementary Figs. 2–5 show that venetoclax induces widespread MOMP and sublethal MOMP in parental MOLM-13 cells that result in cell death over time. On the other hand, in the venR-MOLM-13 cells, venetoclax primarily induces sublethal MOMP, but in the majority of these cells, this does not progress toward widespread MOMP and cell death.

The release of cytochrome C in the cytoplasm is associated with caspase activation, which can be probed by cleavage of caspase 3 and PARP [4]. Moreover, caspase activation can lead to DNA damage, which can be probed by S139-phosphorylation of histone H2Ax by using a γH2Ax-specific antibody [5]. As expected, parental MOLM-13 cells exhibited caspase activation, DNA damage, and PARP cleavage in response to venetoclax (Fig. 2c). Interestingly, the venetoclax-treated venR-MOLM-13 cells also exhibited increased caspase activity, DNA damage, and PARP cleavage, but at a lower level than the venetoclax-treated parental cells (Fig. 2c). Next, to investigate if caspase activity is required for venetoclax-induced sublethal apoptotic signaling and DNA damage in venR cells, we treated venR-MOLM-13 cells with venetoclax and the pan-caspase inhibitor Q-VD-OPh as single agents and in combination for 24 h. Q-VD-OPh significantly inhibited venetoclax-induced sublethal apoptotic signaling and DNA damage (Fig. 2d, e). Together these results argue that in venR-MOLM-13 cells, venetoclax induces caspase-dependent sublethal apoptotic signaling and DNA damage.

Although venetoclax induces sublethal apoptotic signaling and DNA damage in venR-MOLM-13 cells, the majority of these cells survive and continue to proliferate (Supplementary Fig. 3b–d). We, therefore, hypothesized that inhibition of DNA repair in combination with venetoclax could synergistically induce cell death in venR-MOLM-13 cells. To explore this, we treated venR-MOLM-13 cells with 132 anti-cancer compounds as single agents and in combination with 100 nM venetoclax (Supplementary Table 1), and cell viability was measured after 72 h. The drug response was calculated as a drug sensitivity score as described previously [12, 13]. In alignment with our hypothesis, we observed that sensitivity to the PARP inhibitors olaparib and rucaparib increased in combination with venetoclax (Supplementary Fig. 6a, b, Supplementary Table 1). As expected, we also detected a striking sensitization to the MCL1 inhibitor S63845 in the presence of venetoclax (Supplementary Table 1). As the latter has a well-known preclinical synergy in AML cells [14, 15], but is likely a highly challenging clinical combination [15, 16], we did not evaluate it further.

**Fig. 3:**
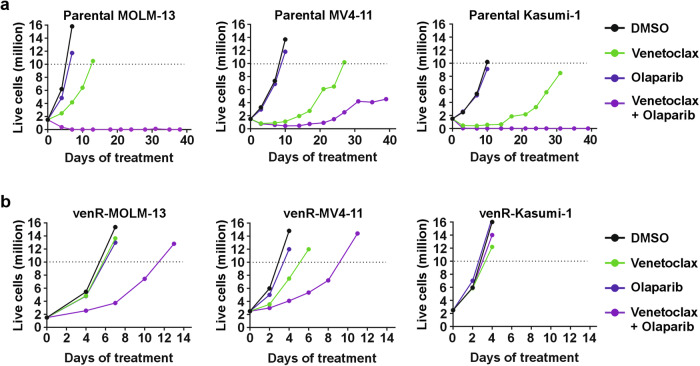
PARP inhibition delays AML cells acquired resistance to venetoclax. The total number of live cells (in millions) at indicated timepoints in **a** parental cell lines and **b** venR-cell lines. The cells were treated with DMSO, 100 nM venetoclax, and 1 µM olaparib as single agents and in combination, with one exception that parental MOLM-13 and venR-MOLM-13 were treated with 50 nM venetoclax for the first 3 days. The total number of live cells was counted after every 2–3 days using trypan blue staining and a Countess II automated cell counter.

To probe for the long-term effects of drug treatment and the emergence of acquired drug resistance, we used a cell-based time-to-progression assay [17]. We treated parental and venR AML cell lines with venetoclax and PARPi, as single agents and in combination for 40 days, where the drugs were replenished, and live cell numbers were counted every 2–3 days. The parental AML cell lines were intrinsically resistant to PARPi alone, and despite the initial sensitivity to venetoclax, these cells acquired resistance to venetoclax within a few weeks (Fig. 3a). Strikingly, PARPi in combination with venetoclax delayed or prevented the acquired resistance of parental AML cell lines to venetoclax (Fig. 3a). In the venR-cells, on the other hand, the venetoclax/PARPi combination only resulted in short-term growth inhibition (Fig. 3b). This indicates that venetoclax-resistant AML cells can quickly acquire resistance to the combination of venetoclax and PARPi.

**Fig. 4:**
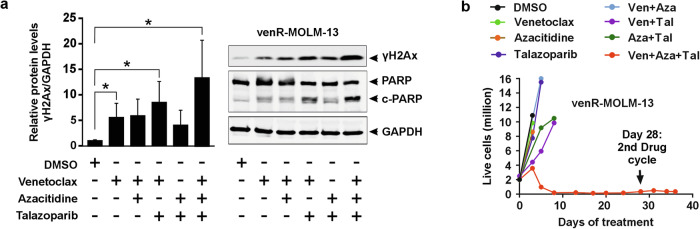
Triple combination of venetoclax, azacitidine, and PARPi induces increased DNA damage and cell death in venetoclax-resistant AML cells. **a** Quantification of γH2Ax when normalized to GAPDH. The immunoblot showing the expression of γH2Ax, PARP (non-cleaved), c-PARP (cleaved-PARP), and GAPDH in the venR-MOLM-13 cells analyzed at 96 h post-drug treatment. The quantification is mean ± s.d. from three independent experiments, and one representative immunoblot is shown. Data were compared using a student’s *t*-test (two-tailed unpaired) to determine statistical significance such that *p* ≤ 0.05 = *. **b** The total number of live cells (in millions) at indicated timepoints. The venR-MOLM-13 cells were treated with DMSO, 100 nM venetoclax (Ven), 100 nM azacitidine (Aza), and 20 nM talazoparib (Tal) as single agents and in combination. The 28-day treatment cycle included daily treatment with all these drugs for the first 7 days, after which azacitidine was discontinued while venetoclax and talazoparib were continued 5 days a week.

To overcome the resistance of venR cells to the venetoclax/PARPi combination, we added azacitidine to the venetoclax/PARPi combination, as azacitidine is clinically relevant, and earlier studies have shown that the combination of PARPi and azacitidine can sensitize AML cells by synergistic induction of DNA damage [10]. Here we used talazoparib to inhibit PARP, as talazoparib is ~100-fold more potent than olaparib and rucaparib to trap PARP-DNA complexes that could lead to increased DNA damage and cytotoxicity [18]. We treated venR-MOLM-13 cells with different combinations of venetoclax, azacitidine, and PARPi, and examined the induction of DNA damage after 96 h. Venetoclax alone induced modest DNA damage, which was increased with the venetoclax/PARPi combination treatment (Fig. 4a). Notably, the DNA damage was further enhanced by the triple combination (Fig. 4a). In long-term cell cultures, the venR cell lines were resistant to single agent or double drug combinations of venetoclax, azacitidine and PARPi (Fig. 4b, Supplementary Fig. 6c). The venetoclax/azacitidine/PARPi triple combination, on the other hand, effectively eradicated or significantly delayed the acquired resistance of venR-cells (Fig. 4b, Supplementary Fig. 6c).

**Fig. 5:**
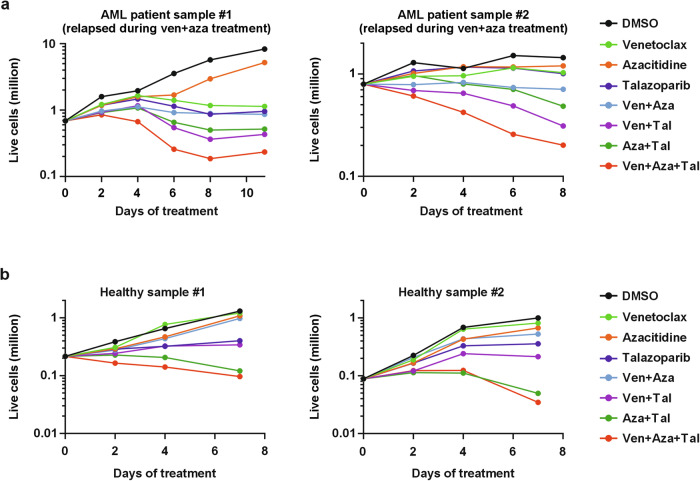
The triple combination of venetoclax, azacitidine, and PARPi more effectively kills cells of AML patients than healthy bone marrow cells from healthy donors. **a**, **b** The total number of live cells (in millions) at indicated timepoints in **a** primary AML samples obtained from patients relapsing under venetoclax-azacitidine treatment and **b** bone marrow samples obtained from healthy donors. The cells were treated with DMSO, 100 nM venetoclax, 100 nM azacitidine, and 20 nM talazoparib as single agents and in combination. The cells were treated with venetoclax and PARPi every other day, while azacitidine was added daily for the first 5 days, after which azacitidine was discontinued.

Finally, we explored the effect of double and triple drug combinations in primary leukemic cells from four patients (Supplementary Table 2) whose AML had relapsed during venetoclax-azacitidine treatment and in bone marrow cells from two healthy donors. In samples from the AML patients the triple combination of venetoclax/azacitidine/PARPi (talazoparib or olaparib) was twice more effective at killing AML cells than the double combination of azacitidine/PARPi (talazoparib or olaparib) (Fig. 5a, Supplementary Fig. 7a). However, in healthy bone marrow samples the double combination of azacitidine/talazoparib or triple combination of venetoclax/azacitidine/talazoparib had similar growth suppressing effects on total cells or specifically on the CD34+ hematopoietic stem and progenitor cells (Fig. 5b, Supplementary Fig. 7b).

**Fig. 6:**
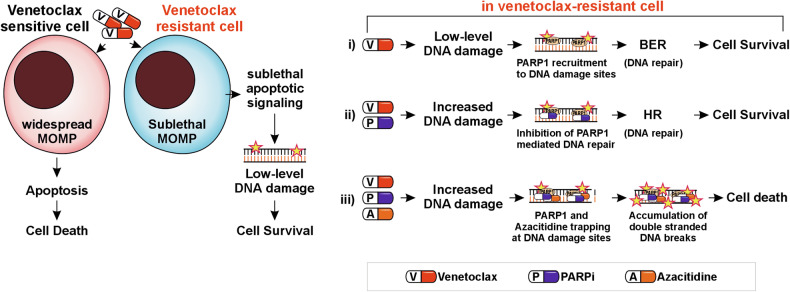
The illustration outlines the proposed mechanistic model. In response to venetoclax, the venetoclax-sensitive cells undergo widespread MOMP, apoptosis, and cell death. In contrast, the venetoclax-resistant cells undergo sublethal MOMP, sublethal apoptotic signaling, and low-level DNA damage, with the majority of these cells surviving. We speculate that, in venR cells, (i) venetoclax alone induces DNA damage that can be repaired by PARP-dependent base excision repair (BER) pathway resulting in cell survival, (ii) combining PARPi with venetoclax inhibits PARP-mediated DNA repair and increases venetoclax-induced DNA damage, however, the cells survive likely due to DNA repair by homologous recombination (HR) pathway, (iii) the addition of azacitidine to venetoclax/PARPi combination amplifies the venetoclax-induced DNA damage likely due to trapping of PARP1 and azacitidine at the DNA damage sites, which results in induction of cytotoxic double-stranded DNA breaks and cell death.

In summary, we uncover a novel vulnerability in venetoclax-resistant AML cells that can be exploited to overcome their resistance. Although venetoclax-resistant AML cells survive and expand in the presence of venetoclax, they undergo a sublethal degree of MOMP, resulting in sublethal caspase activation, sublethal apoptotic signaling, and DNA damage. We show that inhibiting DNA repair by combining PARPi and azacitidine with venetoclax induces cell death in AML cells with acquired venetoclax resistance, likely by enhancing the modest DNA damage induced by venetoclax to irreparable levels of double-stranded DNA breaks (Fig. 6). Therefore, the venetoclax/azacitidine/PARPi combination is a powerful approach for overcoming acquired venetoclax resistance in AML.

## Discussion

BCL2, MCL1, and BCL-xL promote cell survival such that a single cell may depend on one or more of these proteins to prevent induction of apoptosis. Venetoclax specifically inhibits BCL2 and induces apoptosis in cells dependent on BCL2 for their survival [15, 19]. Resistance to venetoclax has been associated with multiple mechanisms, such as decreased dependency on BCL2 and/or dependency on other anti-apoptotic proteins [15, 19], downregulation of proapoptotic protein BAK [20], altered cell metabolism [21, 22], and altered mitochondrial structure [23]. In our study, the derivative AML cell lines that became resistant to venetoclax after long-term exposure showed decreased BCL2 dependency while retaining sensitivity to MCL1i and/or acquired sensitivity to BCL-xLi. Notably, the protein expression and drug response data suggest that the mechanism of acquired resistance to venetoclax in these cell lines could be distinct.

Remarkably, we discovered that venetoclax induces caspase-dependent sublethal apoptotic signaling in venR-MOLM-13 cells that do not translate into cell death but elicit DNA damage. Sublethal apoptotic signaling can result from sublethal MOMP, which occurs when only a fraction of mitochondria in a cell undergoes MOMP (minority MOMP) or a few unprimed mitochondria in a cell repopulate (incomplete MOMP), which may result in cell survival [5–7]. The translocation of cytochrome C from mitochondria to the cytoplasm is an indication of MOMP, however, we did not investigate the extent of MOMP occurring at the level of individual mitochondria. Sublethal MOMP may result in caspase activation and caspase-mediated DNA damage, genomic instability, cellular transformation, and tumorigenesis [5]. Importantly, low-level genotoxic damage can elicit rapid DNA repair responses [24], which, in turn, create a window of opportunity for additional chemical intervention and potentially synthetic lethality. In line with this hypothesis, we observed that PARP inhibition sensitizes venR-AML cells to venetoclax.

While the finding that PARPi in combination with venetoclax induces a strong inhibitory effect in venR cells was promising, we also observed that venR cells exposed to the PARPi/venetoclax combination eventually recovered and resumed proliferation even in the continuous presence of both drugs. DNA damage by venetoclax and persistent PARP inhibition by PARPi could lead to single-stranded DNA breaks that, when unrepaired, can evolve to double-stranded DNA breaks (DSBs), which require homologous recombination (HR) mediated DNA repair [25, 26]. The defects in HR-mediated DNA repair, as seen in BRCA-mutated cancers, cause synthetic lethality in PARPi [8]. In AML, however, BRCA mutations are very rare [8, 27], and we speculate that the venR cells exposed to venetoclax/PARPi recover because of HR-dependent DNA repair.

PARPi blocks PARP enzyme activity and traps PARP proteins at DNA breaks, thereby preventing PARP-mediated DNA repair [8, 9, 26]. It is known that PARPi vary in their ability to trap PARP at sites of DNA damage, i.e., talazoparib is >100 times more potent as a PARP trapper than olaparib and rucaparib and that PARPi toxicity correlates with its ability to trap PARP at DNA damage sites [18, 26]. At the same time, it has been shown that hypomethylating agents azacitidine and decitabine, which are used in the clinics in combination with venetoclax for the treatment of de novo AML ineligible for intensive chemotherapy, synergize with PARPi to increase PARP trapping at DNA breaks resulting in increased DNA damage and cell death [10]. We therefore hypothesized that azacitidine or decitabine, as part of the clinical AML treatment regimen with venetoclax would synergize with PARP-inhibition in venR cells. Indeed, the triple combination of venetoclax/azacitidine/PARPi induced cell death and deep responses in venR AML cell lines and primary AML samples from patients who relapsed under venetoclax and azacitidine treatment. The triple combination of venetoclax/azacitidine/PARPi was twice as effective at killing AML cells than the double combination of azacitidine/PARPi, while their effect on CD34+ cells from healthy individuals was similar. The double combination of decitabine and talazoparib is safely tolerated by AML patients [11]. Moreover, despite the neutropenia and thrombocytopenia commonly observed after venetoclax/azacitidine or venetoclax/decitabine treatment [1, 2], the triplet therapy of venetoclax/azacitidine in combination with either FLT3i or IDHi were well tolerated by patients with myeloid malignancies [28, 29]. Therefore, a separate dose escalation clinical study should be designed to identify optimal doses and a treatment schedule for venetoclax/azacitidine/PARPi combination that provides clinical benefit with limited or manageable toxicities.

Although venetoclax has benefited AML patients who are ineligible for high-dose chemotherapy, the duration of response can be short as patients eventually develop resistance and relapse. In this study, we discovered a novel vulnerability in venetoclax-resistant AML cells in response to venetoclax that can be exploited by the triple drug combination of venetoclax/azacitidine/PARPi to overcome acquired resistance to venetoclax. This triple combination could be an effective alternative for AML patients relapsing or refractory to venetoclax/azacitidine treatment and merits future clinical validation.

## Materials and methods

### Cell lines and cell culture

The MOLM-13, Kasumi-1 and MV4-11 AML cell lines were obtained from Deutsche Sammlung von Mikroorganismen und Zellkulturen (DSMZ, Germany) and cultured in RPMI-1640 medium supplemented with 10–20% fetal bovine serum (FBS), 2 mM L-glutamine, penicillin (100 U/mL), and streptomycin (100 μg/mL), at 37 °C and 5% CO_2_. The cell lines were authenticated using the Promega GenePrint24 System (Promega, WI, USA). We used the MycoAlert PLUS Mycoplasma Detection Kit (Lonza, Switzerland) according to manufacturer’s instructions to detect and verify that the cell lines were free of mycoplasma infection.

### Chemical compounds

The chemical compounds were purchased from MedChemExpress (NJ, USA): venetoclax (#HY-15531), S63845 (#HY-100741), A-1331852 (#HY-19741), olaparib (#HY-10162), talazoparib (#HY-16106), azacitidine (#HY-10586), rucaparib (#HY-10617A) and Q-VD-Oph (#HY-12305), and venetoclax (#CT-A199, ChemieTek, IN, USA).

### Venetoclax-resistant AML cell lines

To generate venetoclax-resistant (venR) cell lines, MOLM-13, Kasumi-1, and MV4-11 cells were treated with increasing concentrations of venetoclax (ranging from 12.5–1000 nM). The drug concentration was doubled every 2 days. The cells that continued to proliferate in the presence of venetoclax at 1000 nM were used as venR-cells.

### Drug sensitivity testing

The parental MOLM-13 and venR-MOLM-13 cells were added to 384-well plates (Corning, NJ, USA) containing chemical compounds at five different concentrations in ten-fold dilution steps (Supplementary Table 1). For drug combinations, venetoclax was added at a fixed dose of 100 nM. After 72 h incubation at 37 °C and 5% CO_2_, cell viability was measured with the CellTiter-Glo reagent (Promega), using a PHERAstar FS plate reader (BMG LABTECH, Germany). For analysis, the DMSO-treated wells were used as negative controls. The drug sensitivity score (DSS) [12], which is a measure based on the area under the dose–response curve was calculated by fitting the four-parameter dose–response curve using the Breeze Pipeline online tool [13].

### Flow cytometry

The cells were stained for markers of viability (DRAQ7; #424001, BioLegend, CA, USA) and apoptosis (Annexin V-FITC; #556420, BD Biosciences, NJ, USA) and counted using an iQue3 flow cytometer (Sartorius, Germany).

### Cell fate tracking after venetoclax exposure (Annexin V staining, cell sorting, colony formation assay, and cell proliferation assay)

The venR-MOLM-13 cells were treated with 100 nM venetoclax for 48 h, after which the cells were stained with Annexin V-FITC as per the manufacturer’s instructions (#560931, BD Biosciences). The cells were analyzed for Annexin V staining using the BD FACSDiscover S8 spectral flow cytometer, and using a cell sorter, we sorted four cell fractions (P1–P4) based on their Annexin V signal intensity such that P1 < P2 < P3 < P4 for Annexin V signal intensity. After sorting, viable cells were counted using trypan blue staining with a Countess II automated cell counter (Thermo Fisher, MA, USA).

For the colony formation assay, the cell suspension (3000 live cells in 300 µL growth medium) was added to 3 mL MethoCult H4236 (#04236, StemCell Technologies, Canada) supplemented with 20% FBS. One mL of this suspension was dispensed by MethoCult syringes (#28110 and #28230, StemCell Technologies) into each well of non-cell-culture-treated 6-well plates (#3736, Corning). Plates were kept on damp paper towels in square polystyrene dishes (#431111, Corning) for 7 days at 37 °C in 5% CO_2_. Whole wells were imaged on an Agilent BioTek Cytation 5 Cell Imaging Multi-Mode Reader (Agilent, CA, USA). Cell colonies were detected and counted using Gen 3.11 software using the following settings: mask 85% of the whole well, accepted colony size <500 µm. Colony counts were multiplied by 1.1781 to obtain total colony numbers per well.

To follow cell proliferation, 50,000 sorted cells were seeded in a well of a 48-well plate (#351178, Corning). Cell proliferation was assessed by counting the viable cell numbers after 2-, 5-, and 7-days using trypan blue staining with a Countess II automated cell counter (Thermo Fisher).

### Cytochrome C flow cytometry assay

Parental and venR-MOLM-13 cells were plated on a v-bottom 96-well plate (#249935, Thermo Fisher) (60,000 cells/well in 100 µL) and were treated with 100 nM venetoclax or DMSO for 4 and 24 h. Cells were cultured at 37 °C and 5% CO_2_. The cells were stained using the BD Pharmingen Transcription Factor Buffer Set (#562574, BD Pharmingen, MA, USA) according to the manufacturer’s instructions. In short, cells were stained with the viability dye eFluor780 (1:1000 dilution) (#65-0865-18, Thermo Fisher) for 30 min at 4 °C, followed by fixation and permeabilization for 45 min at 4 °C and intracellular staining with the cytochrome C—VioBB515 antibody (1:50 dilution) (#130-130-881, Miltenyi, Germany) for 45 min at 4 °C. Data were acquired using an iQue Screener PLUS flow cytometer (Sartorius) and analyzed with the ForeCyt software (Sartorius). For data analysis, the median fluorescence intensity (MFI) was used.

### Cell fractionation

The cells were treated with DMSO and 100 nM venetoclax for 4 h, and the cytoplasmic and mitochondrial cell fractions were extracted using a cell fractionation kit (#ab109719, Abcam, United Kingdom) according to the manufacturer’s instructions. The cytochrome C apoptosis WB antibody cocktail (#ab110415, Abcam) was used to detect cytochrome C, glyceraldehyde-3-phosphodehydrogenase (GAPDH) as a cytoplasmic marker, and pyruvate dehydrogenase subunit E1-alpha (PDHE1-α) and ATP synthase subunit alpha (CV-α) as mitochondrial markers by WB analysis.

### Incucyte live cell imaging

The cell medium was supplemented with caspase-3/7 green dye (#4440, Sartorius) and Cytotox red dye (# 4632, Sartorius). The parental MOLM-13 and venR-MOLM-13 cells were treated with DMSO, venetoclax, or S63845 and were imaged using an Incucyte live cell imaging system for 72 h (Sartorius). The mean fluorescence intensity (MFI) for caspase-3/7 green dye and celltox red dye were calculated using Incucyte analysis software.

### Time-to-progression assay

The cell lines were treated with venetoclax, azacitidine, and either olaparib or talazoparib as single agents or in combination. The drugs were administered daily for the first 7 days, after which azacitidine was discontinued, and only venetoclax and olaparib or talazoparib were added 5 days per week. A full medium change was performed with each drug replenishment, and the total number of live cells was counted using trypan blue staining with a Countess II automated cell counter (Thermo Fisher).

### Western Blotting (WB)

Protein lysates were prepared by treating the cells with RIPA buffer (#9806, Cell Signaling Technology, MA, USA), and the total protein concentration was measured using the Pierce BCA protein assay kit (#23225, Thermo Fisher). The proteins were separated by SDS-PAGE, transferred to nitrocellulose membranes, and blotted using specific antibodies as detailed below. We used an Odyssey scanner (LI-COR Biosciences, NE, USA) for the acquisition of the signals and Image Studio Lite software for the quantification of the blots. The antibodies used were anti-BCL2 (#4223, Cell Signaling Technology), anti-MCL1 (#5453, Cell Signaling Technology), anti-BCL-xL (#2764, Cell Signaling Technology), anti-β-actin (#A1978, Sigma-Aldrich, USA), anti-phospho-histone-H2Ax (Ser139) (#05-636, Merck-Millipore, MA, USA), anti-cleaved Caspase 3 (#9664, Cell Signaling Technology), anti-GAPDH (#G8795, Sigma-Aldrich), anti-PARP (#9542, Cell Signaling Technology). The secondary antibodies IRDye 800 (#926-32211) and IRDye 680 (#926-32220) were purchased from LI-COR Biosciences. The original Western blots are shown in the Supplementary file.

### Samples from AML patients and healthy donors

The samples from AML patients and healthy donors were collected as bone marrow aspirates or peripheral blood draws after informed consent and using protocols approved by an ethics committee of the Helsinki University Hospital and in accordance with the Declaration of Helsinki. The mononuclear cell fractions were separated by density gradient centrifugation and viably cryopreserved. The AML patient cells were thawed using standard conditions followed by culturing in StemSpan SFEMII (#09655, STEMCELL Technologies, Canada) medium supplemented with the following reagents; 50 ng/mL FLT3L (STEMCELL Technologies #78009), 20 ng/mL IL-6 (PeproTech #200-06, NJ, USA), 10 ng/mL IL-3 (PeproTech #200-03), 20 ng/mL GM-CSF (PeproTech #300-03), 20 ng/mL G-CSF (PeproTech #300-23), 10 ng/mL SCF (PeproTech #300-07), IL-1β (PeproTech #200-01B), as well as 0.5 μM SR1 (MedChemExpress # HY-15001) and 1 μM UM729 (#S7510, Selleckchem, TX, USA). After thawing, the samples were cultured for 3 days to ensure expansion prior to drug treatment. The healthy donor cells were thawed using standard conditions followed by culturing in StemSpan SFEMII (#09655, STEMCELL Technologies) medium supplemented with the following reagents: 20 ng/mL SCF, 20 ng/mL FLT3L and 20 ng/mL TPO (PeproTech #300-18). The cells were cultured for 4 days before starting the drug treatments.

For drug treatment, the AML and healthy cells were cultured in 24-well ultra-low attachment plates (Corning) and treated with 100 nM venetoclax, 100 nM azacitidine, and 20 nM talazoparib as single agents and in combination. To mimic the clinical treatment strategy, azacitidine was discontinued after the first 5 days of treatment, and only venetoclax and talazoparib were continued for the remaining culture period. At the day of medium change, 20 μL cells from each condition were stained for markers of cell viability (DRAQ7; #424001, BioLegend), apoptosis (Annexin V-FITC; #556420, BD Biosciences), CD34 (#345804, BD Biosciences) and counted using an iQue3 flow cytometer (Sartorius). The total number of live cells in the wells was calculated by gating DRAQ7 negative and Annexin V negative cells. The CD34+ cells were gated from the total live cell population.

AML patient samples #3 and #4 were treated with 300 nM venetoclax, 300 nM azacitidine, and 1 µM olaparib as single agents and in combinations. The total number of live cells was counted using trypan blue staining with a Countess II automated cell counter (Thermo Fisher).

### Statistical analysis

Mean, standard deviation (s.d.), and statistical significance were calculated with GraphPad version 9 (GraphPad Software Inc., USA). Data were compared using a student’s *t*-test (a two-tailed unpaired, a two-tailed paired, or a two-tailed one sample) to determine statistical significance such that *p* ≤ 0.05 = * and *p* ≤ 0.01 = **.

## Supplementary information

Supplementary file

Supplementary Table 1

Supplementary Table 2

Supplementary Figure 1

Supplementary Figure 2

Supplementary Figure 3

Supplementary Figure 4

Supplementary Figure 5

Supplementary Figure 6

Supplementary Figure 7

## Data Availability

All data generated or analyzed during this study are included in this published article and its Supplementary Information files.
