# Supplementary material for: Venetoclax triggers sublethal apoptotic signaling in venetoclax-resistant acute myeloid leukemia cells and induces vulnerability to PARP inhibition and azacitidine
Source: Cell Death Dis. 2024 Oct 16;15(10):750. doi: 10.1038/s41419-024-07140-4 (PMC11484809; doi:10.1038/s41419-024-07140-4)
Supplement: Supplementary file 1 — Supplementary file [file 41419_2024_7140_MOESM1_ESM.pdf]

Venetoclax triggers sub-lethal apoptotic signaling in venetoclax-resistant acute myeloid leukemia cells and induces vulnerability to PARP inhibition and azacitidine

Mahesh Tambe[1], Sarah Unterberger[2], Mette C. Kriegbaum[2], Ida Vänttinen[1], Ezgi June Olgac[1], Markus Vähä-Koskela[1], Mika Kontro[1,3,4], Krister Wennerberg[2#], Caroline A. Heckman[1#]

Running Title: PARP inhibition synergizes with venetoclax/aza in AML

Affiliations

[1] Institute for Molecular Medicine Finland (FIMM), Helsinki Institute of Life Science (HiLIFE), iCAN Digital Precision Cancer Medicine Flagship, University of Helsinki, Helsinki, Finland

[2] Biotech Research & Innovation Centre (BRIC), University of Copenhagen, Copenhagen, Denmark

[3] Department of Hematology, Helsinki University Central Hospital Comprehensive Cancer Center, Helsinki, Finland

[4] Foundation for the Finnish Cancer Institute, Helsinki, Finland

[#] **Corresponding authors**

Caroline A. Heckman: caroline.heckman@helsinki.fi

Krister Wennerberg: krister.wennerberg@bric.ku.dk

Supplementary Figure Legends

Supplementary Figure 1: The quantification of BCL2, MCL1 and BCL-xL proteins in parental and venR (MOLM-13, Kasumi-1 and MV4-11) cells normalized to β-actin. The data is mean +/- s.d. from 2-3 independent experiments.

Supplementary Figure 2: (a) Gating strategy for flow cytometry analysis. Cells were gated based on SSC-H/FSC-H, followed by the identification of singlets using FSC-A/FSC-H. Fluorescence intensities for Annexin V and DRAQ7 are shown on the horizontal axis and vertical axis, respectively. **(b, c)** Representative flow cytometry analysis of (b) parental MOLM-13 and (c) venR-MOLM-13 cells treated with DMSO and venetoclax for 0, 4, 24, 48 and 72 h.

Supplementary Figure 3: (a) Gating strategy for sorting the cells. The venR-MOLM-13 cells were treated with venetoclax for 48 h after which the cells were stained with Annexin V, and four cell fractions (P1, P2, P3 and P4) were sorted according to their increasing Annexin V signal intensity such that P1<P2<P3<P4 for Annexin V signal intensity. The R3 channel on the Y-axis is an arbitrary channel used to facilitate gating. **(b)** The venetoclax-treated Annexin V-sorted cell populations P1-P4 and untreated venR-MOLM-13 cells were cultured in normal growth conditions, and their fate was followed by assessing cell viability and live cell numbers at day 2-, 5- and 7. Live cell numbers and cell viability was measured by using trypan blue staining and a Countess II automated cell counter. Data are presented as the mean ± s.d. from 3 independent experiments. **(c, d)** The number of cell colonies and representative images from the colony formation assay of untreated venR-MOLM-13 cells and venetoclax-treated Annexin V sorted cell populations P1-P4. Data are presented as the mean ± s.d. from 3 independent experiments.

Supplementary Figure 4: (a) The representative images of parental MOLM-13 and venR-MOLM-13 cells treated with DMSO, 100 nM venetoclax (BCL2i) and 100 nM S-63845 (MCL1i) for 72 h. The green signals represent cells with caspase-3/7 activation whereas red signals represent dead cells**. (b)** The graph shows mean fluorescence intensity (MFI) of caspase-3/7 green dye measured from each cell and normalized to DMSO. **(c)** The graph shows mean fluorescence intensity (MFI) of cytotox red dye measured from each cell and normalized to DMSO.

Supplementary Figure 5: (a) Gating strategy for flow cytometry analysis. Cells were gated based on SSC-H/FSC-H, followed by the identification of singlets using FSC-A/FSC-H. Viable cells were gated by using fixable viability dye eFluor™ 780 (ef780) and only the ef780 negative cells (live cells) were included for

further analysis. Live cells were then used to assess the cytochrome C negative (Cyt-C-; left side) and cytochrome C positive (Cyt-C+; right side) fraction, and the fluorescence intensity of cytochrome C in cells treated with DMSO (green) or venetoclax (blue) are plotted against the number of events in an overlay histogram. **(b, c, d)** Parental and venR-MOLM-13 cells were treated with 100 nM venetoclax or DMSO for 4 and 24 h followed by staining of intracellular cytochrome C. (b) The fluorescence intensity of cytochrome C in cells treated with DMSO (green) and venetoclax (blue) is plotted against the number of events in an overlay histogram. Arrows indicate peak shift towards loss of cytochrome C after venetoclax treatment which indicates sublethal MOMP. (c) The percentage of cytochrome C negative cells (cells undergoing widespread MOMP) after 4 and 24 h of DMSO or venetoclax treatment. (d) The median fluorescence intensity (MFI) of cytochrome C positive cells normalized to DMSO after 4 and 24 h venetoclax treatment. The data is from three independent experiments. Data were compared using a two-tailed one sample t-test or with using a two-tailed paired t-test to determine statistical significance such that $p \leq 0.05$ = *.

Supplementary Figure 6: (a-b) Percentage cell viability in venR-MOLM-13 cells in response to (a) olaparib plus DMSO and olaparib plus 100 nM venetoclax; and (b) rucaparib plus DMSO and rucaparib plus 100 nM venetoclax, measured after 72 h of drug treatment using the CellTiter-Glo assay. The corresponding IC50 values are illustrated on the graphs. The data are presented as mean +/- s.d. from three independent experiments. **(c)** Quantification of total number of live cells (in millions) at the indicated timepoints. The venR cells were treated with DMSO, 100 nM venetoclax, 100 nM azacitidine, and 1 µM olaparib as single agents and in combination. The 28-day treatment cycle included daily treatment with all drugs for the first 7 days, after which azacitidine was discontinued while venetoclax and PARPi were added daily for 5-days a week.

Supplementary Figure 7: (a) The total number of live cells (in millions) at indicated timepoints in primary AML samples obtained from patients relapsing under venetoclax-azacitidine treatment. The cells were treated with DMSO, 300 nM venetoclax, 300 nM azacitidine, and 1 µM olaparib as single agents and in combination**.** The cells were treated with venetoclax and olaparib every other day, while azacitidine was added daily for the first 5 days, after which azacitidine was discontinued. **(b)** The total number of CD34+ cells at indicated

timepoints in bone marrow cells obtained from healthy donors. The bone marrow cells were treated with DMSO, 100 nM venetoclax, 100 nM azacitidine, and 20 nM talazoparib as single agents and in combination**.** The cells were treated with venetoclax and talzoparib every other day, while azacitidine was added daily for the first 5 days, after which azacitidine was discontinued.

Original Western Blots

Figure 1 d, e, f

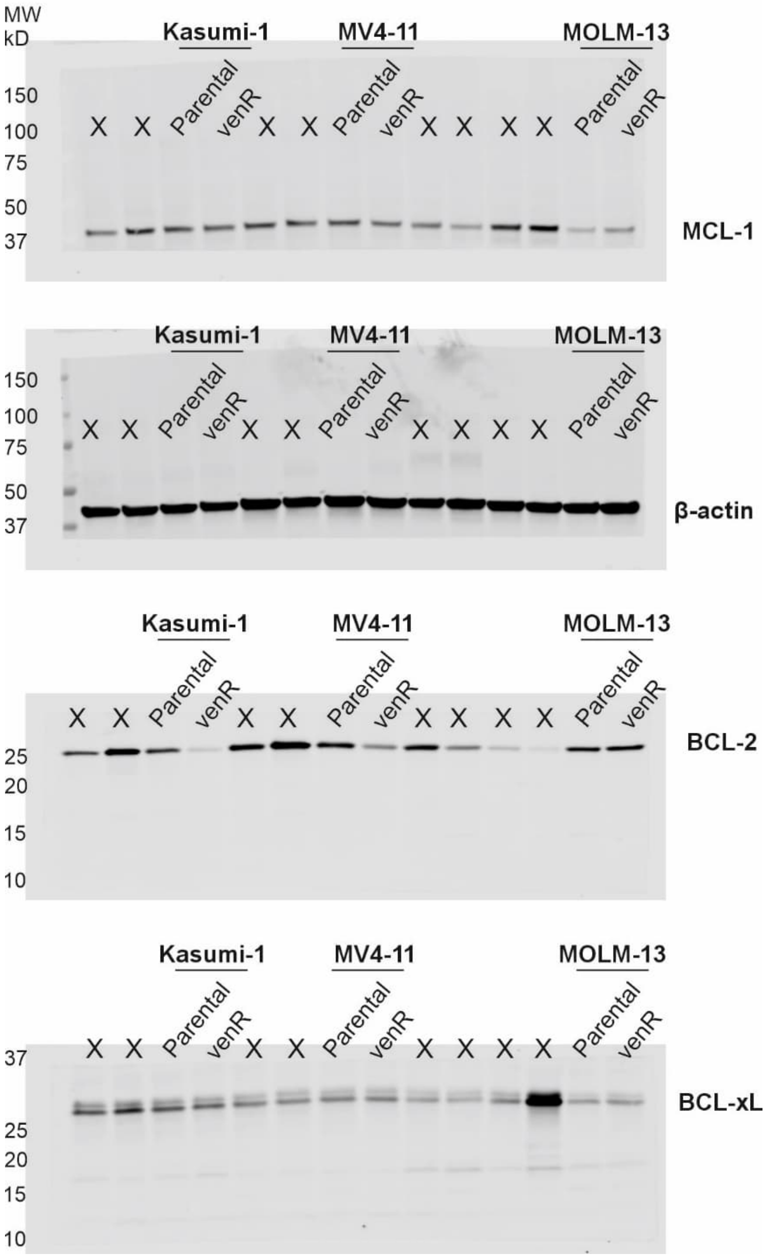

Figure 2b

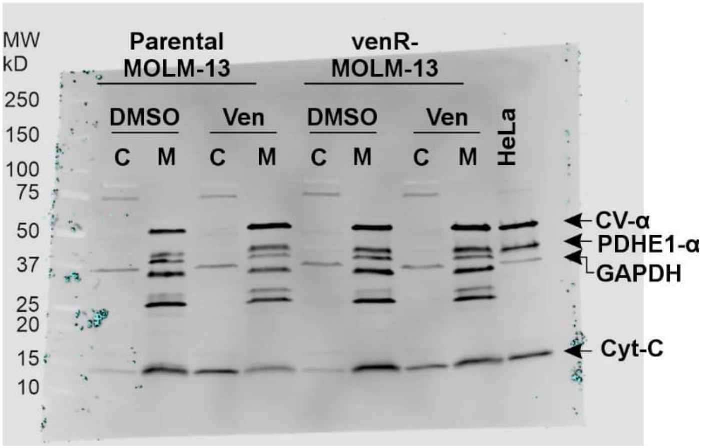

Figure 2c

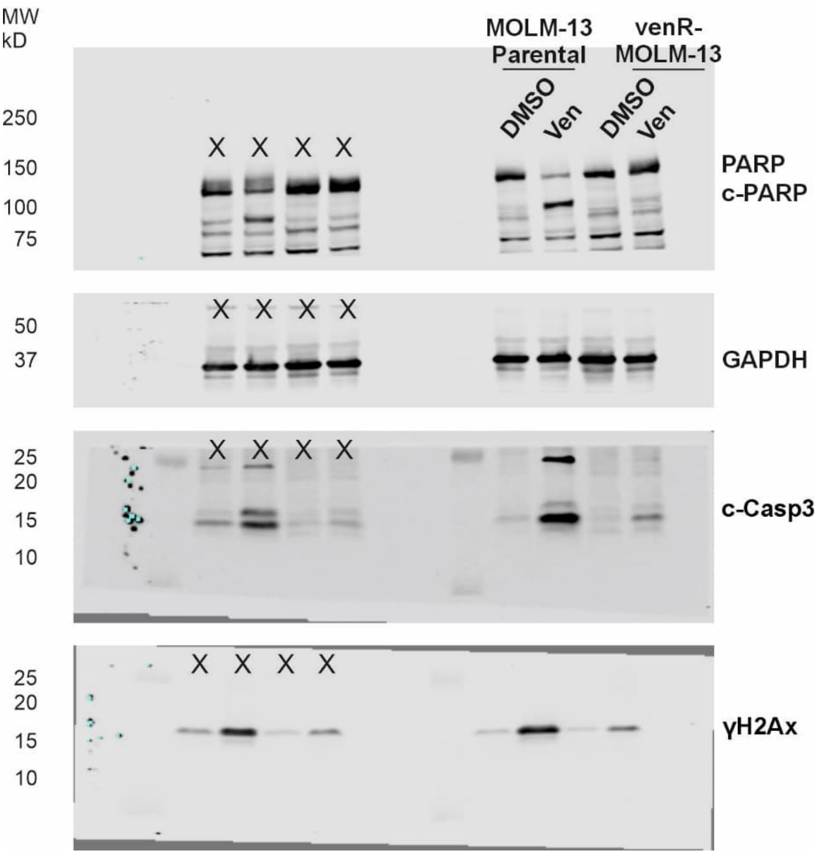

Figure 2e

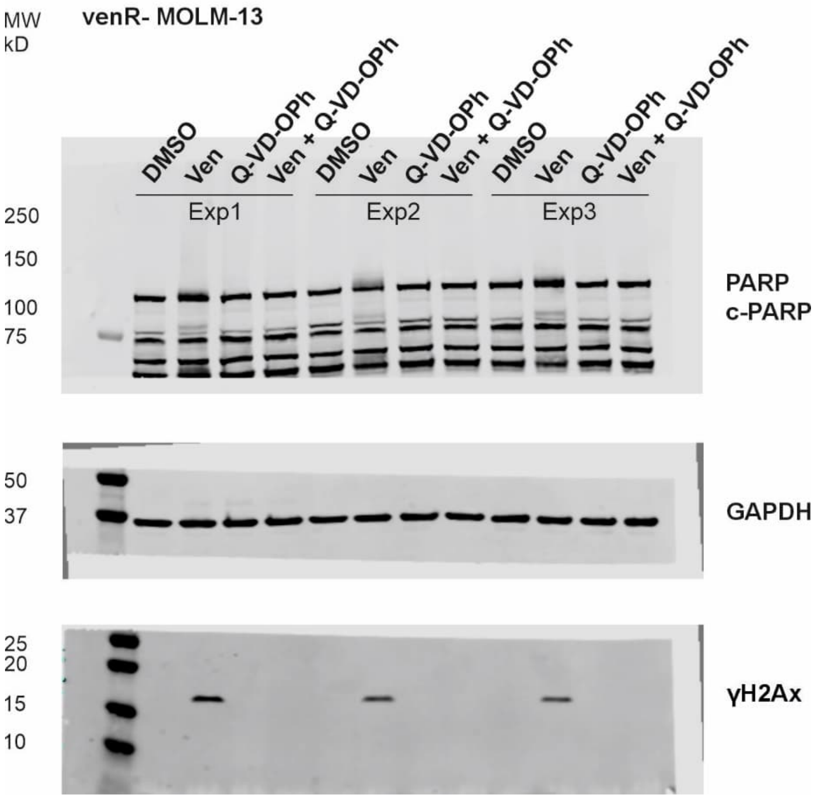
MW
kD
venR- MOLM-13
DMSO
Ven
Q-VD-OPh
Ven + Q-VD-OPh
Exp1
Exp2
Exp3
250
150
100
75
PARP
c-PARP
50
37
GAPDH
25
20
15
10
γH2Ax

Figure 4a

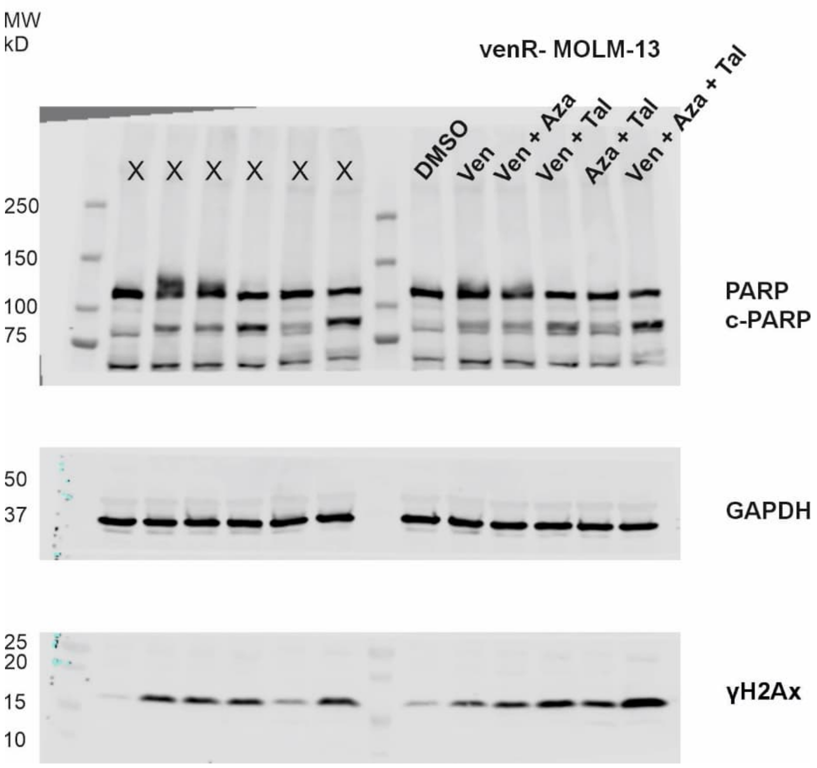
MW
kD
venR- MOLM-13
X X X X X X
DMSO
Ven
Ven + Aza
Ven + Tal
Aza + Tal
Ven + Aza + Tal
250
150
100
75
PARP
c-PARP
50
37
GAPDH
25
20
15
10
γH2Ax
