# Supplementary material for: Venetoclax triggers sublethal apoptotic signaling in venetoclax-resistant acute myeloid leukemia cells and induces vulnerability to PARP inhibition and azacitidine
Source: Cell Death Dis. 2024 Oct 16;15(10):750. doi: 10.1038/s41419-024-07140-4 (PMC11484809; doi:10.1038/s41419-024-07140-4)
Supplement: Supplementary file 2 — Supplementary Table 1 [file 41419_2024_7140_MOESM2_ESM.docx]

**Supplementary Table 1**: Cell viability as indicated by drug sensitivity scores (DSS) for the indicated compounds as single agents and in combination with venetoclax (100 nM) in venR-MOLM13 cells measured after 72 h of drug treatment. Higher DSS indicates increased sensitivity.

| **DRUG NAME** | **Single drug (Drug sensitivity score)** | **Drug + venetoclax (Drug sensitivity score)** | **[Drug+venetoclax]- [single drug] (Drug Sensitivity Score)** |
| --- | --- | --- | --- |
| S63845 | 9.3 | 28.6 | 19.3 |
| **Olaparib** | 5.4 | 10 | 4.6 |
| A-1331852 | 0.5 | 4.6 | 4.1 |
| Mitoxantrone | 16.4 | 20.1 | 3.7 |
| LY-3009120 | 18.2 | 21.9 | 3.7 |
| **Rucaparib** | 4.4 | 8 | 3.6 |
| Omacetaxine | 38.1 | 41.4 | 3.3 |
| TIC10 | 15.2 | 18.3 | 3.1 |
| AZD1152-HQPA | 22.3 | 25.3 | 3 |
| Valproic acid | 0 | 2.8 | 2.8 |
| Alisertib | 24.6 | 27.3 | 2.7 |
| Etoposide | 25.5 | 28.2 | 2.7 |
| Imatinib | 0.6 | 3.2 | 2.6 |
| Alvocidib | 26.1 | 28.6 | 2.5 |
| Idasanutlin | 23.9 | 26.3 | 2.4 |
| Birinapant | 7.3 | 9.6 | 2.3 |
| Idarubicin | 29.6 | 31.8 | 2.2 |
| Axitinib | 13.3 | 15.5 | 2.2 |
| PTC-209 | 12.9 | 15.1 | 2.2 |
| Vorinostat | 15.4 | 17.5 | 2.1 |
| Molibresib | 14.5 | 16.5 | 2 |
| Adavosertib | 22.3 | 24.1 | 1.8 |
| SCH772984 | 16.6 | 18.3 | 1.7 |
| Topotecan | 33.6 | 35.3 | 1.7 |
| Vincristine | 37.5 | 39.1 | 1.6 |
| Tosedostat | 12.7 | 14.3 | 1.6 |
| Selinexor | 24.5 | 26.1 | 1.6 |
| NVP-LCL161 | 5.8 | 7.4 | 1.6 |
| Ceritinib | 1.5 | 3.1 | 1.6 |
| Bosutinib | 13.6 | 15.1 | 1.5 |
| Bemcentinib | 20 | 21.4 | 1.4 |
| Ulixertinib | 9.5 | 10.8 | 1.3 |
| Sunitinib | 26.1 | 27.4 | 1.3 |
| Taselisib | 10.6 | 11.8 | 1.2 |
| Chloroquine | 10.8 | 12 | 1.2 |
| Sapanisertib | 20.4 | 21.5 | 1.1 |
| Rocilinostat | 9.7 | 10.8 | 1.1 |
| Tazemetostat | 0 | 1.1 | 1.1 |
| Volasertib | 23.6 | 24.7 | 1.1 |
| Azacitidine | 0 | 1 | 1 |
| Cobimetinib | 13.4 | 14.4 | 1 |
| Belinostat | 19.1 | 20 | 0.9 |
| VS-4718 | 21.3 | 22.2 | 0.9 |
| Fludarabine | 9 | 9.8 | 0.8 |
| Cytarabine+Idarubicin | 27.6 | 28.4 | 0.8 |
| Gilteritinib | 21.4 | 22.1 | 0.7 |
| AZD8186 | 4.6 | 5.3 | 0.7 |
| Sorafenib | 25.7 | 26.4 | 0.7 |
| Navtemadlin | 8.8 | 9.5 | 0.7 |
| Opaganib | 8.5 | 9.2 | 0.7 |
| Ponatinib | 35.5 | 36.1 | 0.6 |
| Crenolanib | 41.5 | 42.1 | 0.6 |
| Birabresib | 21.4 | 22 | 0.6 |
| Ruxolitinib | 6 | 6.6 | 0.6 |
| Momelotinib | 20.6 | 21.2 | 0.6 |
| Cytarabine | 23.6 | 24.1 | 0.5 |
| Erlotinib | 8.7 | 9.1 | 0.4 |
| Quizartinib | 33.5 | 33.9 | 0.4 |
| Nintedanib | 34.5 | 34.9 | 0.4 |
| Pevonedistat | 26.8 | 27.2 | 0.4 |
| Bortezomib | 31.4 | 31.7 | 0.3 |
| Panobinostat | 25.8 | 26.1 | 0.3 |
| Gemcitabine | 28.5 | 28.8 | 0.3 |
| Gedatolisib | 22.8 | 23.1 | 0.3 |
| Alectinib | 5.8 | 6.1 | 0.3 |
| Tipifarnib | 19.6 | 19.9 | 0.3 |
| Paclitaxel | 32.3 | 32.5 | 0.2 |
| Entospletinib | 10.2 | 10.4 | 0.2 |
| Ralimetinib | 5.6 | 5.8 | 0.2 |
| Vistusertib | 24.2 | 24.4 | 0.2 |
| Copanlisib | 13.8 | 14 | 0.2 |
| Dasatinib | 8.7 | 8.8 | 0.1 |
| Erdafitinib | 0.1 | 0.2 | 0.1 |
| Duvelisib | 4.5 | 4.6 | 0.1 |
| Metformin | 0 | 0 | 0 |
| Anagrelide | 0 | 0 | 0 |
| Dexamethasone | 0 | 0 | 0 |
| Celecoxib | 0 | 0 | 0 |
| Hydroxyurea | 0 | 0 | 0 |
| Pravastatin | 0 | 0 | 0 |
| Nilotinib | 0 | 0 | 0 |
| Vemurafenib | 2.3 | 2.3 | 0 |
| Vismodegib | 0 | 0 | 0 |
| Galunisertib | 0 | 0 | 0 |
| MK-0752 | 0 | 0 | 0 |
| Infigratinib | 0 | 0 | 0 |
| Devimistat | 0 | 0 | 0 |
| Glasdegib | 0 | 0 | 0 |
| Epacadostat | 0 | 0 | 0 |
| BRD7116 | 0 | 0 | 0 |
| Ivosidenib | 0 | 0 | 0 |
| Selonsertib | 0 | 0 | 0 |
| Larotrectinib | 0 | 0 | 0 |
| Brequinar | 23.9 | 23.8 | -0.1 |
| Methylprednisolone | 0.1 | 0 | -0.1 |
| Roxadustat | 0.1 | 0 | -0.1 |
| Venetoclax | 0.1 | 0 | -0.1 |
| Carfilzomib | 30 | 29.9 | -0.1 |
| Midostaurin | 34.5 | 34.4 | -0.1 |
| Dinaciclib | 27.6 | 27.5 | -0.1 |
| Ceralasertib | 20.8 | 20.7 | -0.1 |
| Hydroxyfasudil | 1.8 | 1.6 | -0.2 |
| Tretinoin | 6.2 | 5.9 | -0.3 |
| Regorafenib | 24.5 | 24.2 | -0.3 |
| Filanesib | 30.5 | 30.2 | -0.3 |
| Idelalisib | 8.4 | 7.9 | -0.5 |
| Vidofludimus | 6 | 5.5 | -0.5 |
| Ipatasertib | 7.6 | 7 | -0.6 |
| NVP-SHP099 | 3.3 | 2.7 | -0.6 |
| Amcasertib | 14.5 | 13.9 | -0.6 |
| Onalespib | 24.3 | 23.7 | -0.6 |
| Mepacrine | 21.5 | 20.8 | -0.7 |
| Enasidenib | 0.7 | 0 | -0.7 |
| Ibrutinib | 4.7 | 3.9 | -0.8 |
| Clofarabine | 29.8 | 29 | -0.8 |
| Trametinib | 16.3 | 15.5 | -0.8 |
| Methotrexate | 30.5 | 29.5 | -1 |
| Palbociclib | 20.2 | 19.2 | -1 |
| Pacritinib | 28.9 | 27.9 | -1 |
| Napabucasin | 19.5 | 18.4 | -1.1 |
| Abemaciclib | 18.4 | 17.2 | -1.2 |
| Silmitasertib | 6.3 | 5 | -1.3 |
| Pinometostat | 5.5 | 4.1 | -1.4 |
| Navitoclax | 6.3 | 4.7 | -1.6 |
| Everolimus | 14.5 | 12.9 | -1.6 |
| Lenalidomide | 5.8 | 4.2 | -1.6 |
| GSK2879552 | 10 | 8.4 | -1.6 |
| Afatinib | 1.6 | 0 | -1.6 |
| Alpelisib | 4.8 | 2.6 | -2.2 |
| AZD0156 | 6.6 | 4.3 | -2.3 |
| NVP-PIM447 | 16.7 | 13.8 | -2.9 |
| Pomalidomide | 7.1 | 3.1 | -4 |
