# Supplementary material for: Venetoclax triggers sublethal apoptotic signaling in venetoclax-resistant acute myeloid leukemia cells and induces vulnerability to PARP inhibition and azacitidine
Source: Cell Death Dis. 2024 Oct 16;15(10):750. doi: 10.1038/s41419-024-07140-4 (PMC11484809; doi:10.1038/s41419-024-07140-4)
Supplement: Supplementary file 3 — Supplementary Table 2 [file 41419_2024_7140_MOESM3_ESM.docx]

**Supplementary Table 2**: Characteristics of the AML patients that contributed samples to the study.

|  | **AML patient #1** | **AML patient #2** | **AML patient #3** | **AML patient #4** |
| --- | --- | --- | --- | --- |
| Gender | Female | Male | Female | Male |
| Age at diagnosis | 69 | 80 | 67 | 74 |
| Diagnosis | AML, MRC (antecedent CMML) | AML with minimal maturation | AML, therapy related (MRC) | AML, therapy related (MRC) |
| AML FAB subtype | M2 | M1 | ND | ND |
| Chromosomal Abnormalities (G-stain) | No | No | Del7q | +11, i(22q) |
| Myeloid gene mutations (excluding VUS) | *TET2* (NM_001127208.2)  exon 6: c.3646C>T p.(Arg1216Ter), VAF 46%.  *SRSF2* (NM_003016.4) exon 1: c.284C>A p.(Pro95His), VAF 46%.  *IDH2* (NM_002168.2) exon 4: c.419G>A p.(Arg140Gln), VAF 38%.  *CSF3R* (NM_000760.3) exon 14: c.1853C>T p.(Thr618Ile), VAF 6%  *CALR* (NM_004343.3) exon 9: c.1150_1154dellinsTGTC p. (Asp384Cysfs*46), VAF 5% | *RUNX1* (NM_001754.4) exon 4: c.315C>G p.(His105Gln), VAF 93%.  *SRSF2* (NM_003016.4) exon 1: c.284C>A p.(Pro95His), VAF 50%.  *IDH2* (NM_002168.2) exon 4: c.419G>A p.(Arg140Gln), VAF 45%.  *FLT3* (NM_004119.2) exon 14: c.1793_1794ins18 p. (Tyr597_Glu598insAspGlnProTyrHisTyr, *FLT3*-ITD), VAF 34%. | *NRAS* (NM_002524.4) exon 2: c.35G>A p.(Gly12Asp), VAF 12%.  *IDH1* (NM_005896.3) exon 4: c.394C>T p.(Arg132Cys), VAF 7%.  *WT1* (NM_024426.4) exon 9: c.1390G>A p.(Asp464Asn), VAF 18%.  *WT1* (NM_024426.4) exon 7: c.1138C>G p.(Arg380Gly), VAF 10%.  *WT1* (NM_024426.4) exon7: c.1128dup p.(Thr377Aspfs*8), VAF 7%. | *SRSF2* (NM_003016.4) exon 1: c.284C>A p.(Pro95His), VAF 50%.  *NRAS* (NM_002524.4) exon 2: c.38G>A p.(Gly13Asp), VAF 24%.  *JAK2* (NM_004972.3) exon14: c.1849G>T p.(Val617Phe), VAF 6%. |
| Treatment and clinical response | Four cycles of aza-ven with CRi response but then relapsed | Two cycles of aza-ven with no response | Five cycles of aza-ven with partial response (blasts 40% →5%) | Six cycles of aza-ven with initial tumor lysis and CR → relapse |

ND: not determined
